# Migratory Movements of Pygmy Blue Whales (*Balaenoptera musculus brevicauda*) between Australia and Indonesia as Revealed by Satellite Telemetry

**DOI:** 10.1371/journal.pone.0093578

**Published:** 2014-04-09

**Authors:** Michael C. Double, Virginia Andrews-Goff, K. Curt S. Jenner, Micheline-Nicole Jenner, Sarah M. Laverick, Trevor A. Branch, Nicholas J. Gales

**Affiliations:** 1 Australian Marine Mammal Centre, Australian Antarctic Division, Kingston, Tasmania, Australia; 2 Centre for Whale Research (Western Australia) Inc., Fremantle, Western Australia, Australia; 3 School of Aquatic and Fishery Sciences, University of Washington, Seattle, Washington, United States of America; Institut Pluridisciplinaire Hubert Curien, France

## Abstract

In Australian waters during the austral summer, pygmy blue whales (*Balaenoptera musculus brevicauda)* occur predictably in two distinct feeding areas off western and southern Australia. As with other blue whale subspecies, outside the austral summer their distribution and movements are poorly understood. In order to describe the migratory movements of these whales, we present the satellite telemetry derived movements of eleven individuals tagged off western Australia over two years. Whales were tracked from between 8 and 308 days covering an average distance of 3,009±892 km (mean ± se; range: 832 km–14,101 km) at a rate of 21.94±0.74 km per day (0.09 km–455.80 km/day). Whales were tagged during March and April and ultimately migrated northwards post tag deployment with the exception of a single animal which remained in the vicinity of the Perth Canyon/Naturaliste Plateau for its eight day tracking period. The tagged whales travelled relatively near to the Australian coastline (100.0±1.7 km) until reaching a prominent peninsula in the north-west of the state of Western Australia (North West Cape) after which they travelled offshore (238.0±13.9 km). Whales reached the northern terminus of their migration and potential breeding grounds in Indonesian waters by June. One satellite tag relayed intermittent information to describe aspects of the southern migration from Indonesia with the animal departing around September to arrive in the subtropical frontal zone, south of western Australia in December. Throughout their migratory range, these whales are exposed to impacts associated with industry, fishing and vessel traffic. These movements therefore provide a valuable tool to industry when assessing potential interactions with pygmy blue whales and should be considered by conservation managers and regulators when mitigating impacts of development. This is particularly relevant for this species as it continues to recover from past exploitation.

## Introduction

Two blue whale subspecies are recognised in the Southern Hemisphere – the Antarctic or true blue whale (*B. m. intermedia)* and the pygmy blue whale (*B. m. brevicauda)* [1]. The pygmy subspecies was only described in the early 1960s [2] once Antarctic blue whale stocks had crashed. The vast majority of blue whale catches in the Southern Hemisphere and northern Indian Ocean were carried out prior to the identification of the pygmy blue whale subspecies and as such, the impact of commercial whaling on this subspecies is not well known [3]. However, both Japan (1959/60–1963/64) and the USSR (1962/63–1972/73) ran expeditions capitalising on their recognition [4] and it is thought that almost all historical blue whale catches north of 52°S and between 35°E and 180°E were pygmy blue whales [3], [5]. It is likely therefore that whaling caused a decrease in pygmy blue whale numbers [3] with their recovery potentially slower than that of the Antarctic blue whale [5]. The current conservation status of this subspecies is highly uncertain with the IUCN Red List of Threatened Species listing it as data deficient [6].

The distribution and seasonal movements of pygmy blue whales are also poorly understood [4], [7]. Prey availability clearly influences the distribution of blue whales worldwide with individuals concentrating in areas of high primary and secondary productivity and where dynamic oceanographic processes such as upwelling and frontal meandering occur [4], [7]. At least four populations of pygmy blue whales are known to span the northern Indian Ocean, from Madagascar to the Subantarctic, from Indonesia to western and southern Australia and from New Zealand to the equator [4].

Branch et al. [4] hypothesised that pygmy blue whales occurring in Australian waters migrate between Australia and Indonesia via the western coast of Australia. This hypothesis is supported by acoustic recordings collected off southwestern Australia [7]–[9] with Australian type pygmy blue whale calls increasing between November and June and peaking between February and May. However, Australian type pygmy blue whale calls have also been recorded in the southwest Indian Ocean subtropical frontal zone between January and June [10], [11] and individuals may occupy Antarctic waters during the austral winter [12]. Off southern and western Australia, pygmy blue whales occur predictably in two distinct feeding areas throughout the austral summer [13], [14]. Historically, these were areas where Soviet whalers caught large numbers of pygmy blue whales and where strandings have occurred [4]. Both of these areas are supported by complex seasonal upwelling systems producing high prey densities [13], [14]. For the southern Australian feeding area, animals are only sighted between November and May [15]. Mark-recapture analyses estimate that between 569 and 1147 individuals occur in the feeding area off western Australia [16] and a line-transect survey off the southern coast of Australia estimated 671 (279–1613) individuals in a small area [17]. Genetic analyses indicate that the animals utilising these two feeding grounds are comprised of a single breeding stock [18].

For highly mobile and migratory species with dynamic and poorly understood migration routes, conservation and management remain a challenge [19]. When migratory animals routinely cross international borders, international cooperation is required to implement conservation strategies that utilise information on habitat use and movement patterns [20]. In order to describe the movement and migratory behaviours of pygmy blue whales and to investigate whether pygmy blue whales feeding in Australian waters do indeed migrate into Indonesian waters, we present here the first published satellite telemetry data for pygmy blue whales. Movement information such as this can reveal potential or unrealised impacts, especially for areas where data collection such as acoustic recordings or sightings are not possible. This information is necessary for managers to protect and conserve pygmy blue whales throughout their range and is valuable to industry when assessing the potential impacts of development, shipping and resource extraction.

## Methods

### Ethics Statement

This study was carried out in strict accordance with the approvals and conditions set by the Australian Antarctic Division’s Antarctic Animal Ethics Committee for this project - Australian Antarctic Science project 2941. Additionally, this study was also carried out in strict accordance with the approvals and conditions set by the Western Australian Department of Environment and Conservation Animal Ethics Committee for this project - 30/2008. Fieldwork was undertaken in Commonwealth Waters with the permission of the Australian Government under EPBC permits 2007-006 and 2007-007.

### Satellite Tag Deployment

The satellite tags employed are comprised of a custom-designed, anchor section joined to a stainless steel housing containing a Spot 5 transmitter manufactured by Wildlife Computers (Redmond, Washington, USA). Satellite tags were deployed using a modified version of the Air Rocket Transmitter System (ARTS, Restech) [21] at a pressure of 7.5–10 bar. Retention teeth on a purpose-designed projectile carrier grip to a metal ring fitted at the end of the tag allowing the tag to be fired from the air gun. When the tag makes contact with the whale, the rapid deceleration of the tag/projectile carrier withdraws the retention teeth releasing the projectile carrier. The metal ring then falls off in time to reduce the drag of the tag. The tag is sterilised with ethylene oxide prior to deployment and implants up to a maximum of 290 mm into the skin, blubber, interfacial layers and outer muscle mass of the whale. Retention of the tag is maintained through two actively sprung plates and a circle of passively deployed ‘petals.’ Once the tag is immersed in salt water, the salt water switch activates and the tag begins to transmit locations via the Argos satellite system when at the surface. Tags deployed in 2009 were duty cycled to a six hours on, 18 hours off transmission period at a 30 second repetition rate. In 2011, tags were duty cycled to a four hours on, eight hours off transmission period at a 30 second repetition rate. Each tag was deployed from the bow-sprit of a 5.8 m rigid-hulled inflatable boat at a range of 3–8 m and was positioned high on the body, approximately in line with the pectoral fins.

### Biopsy Collection

Upon tag deployment, a small amount of skin and blubber was simultaneously collected for genetic analyses. These were collected using a biopsy dart fired from a modified.22 Paxarms system [22]. Biopsy samples were stored in 70% ethanol and DNA subsequently extracted using a Tissue DNA purification kit for the Maxwell 16 DNA extraction robot (Promega Corporation). The sex of the tagged whales was determined using a 5′ exonuclease assay of the polymorphisms in the sex-linked Zinc Finger genes as described by Morin et al. [23].

### Argos Data Processing

For each Argos location, at least prior to 2013, an estimated error is calculated when at least four messages are received during a satellite pass. A location class (LC) is assigned to each location based on this estimate of error. LC 3 has an estimated error of <250 m, LC 2 has an estimated error between 250 and 500 m, and LC 1 has an estimated error between 500 and 1500 m. LC 0 has an open ended error of >1500 m whilst LC A and B have no accuracy estimation and LC Z is an invalid location. Argos locations were filtered using an algorithm based on swimming speed, distance between successive locations, and turning angles [24] to remove unlikely position estimates (default thresholds – speed of 2 ms^−1^, spike angles of 15° and 25°, spike lengths of 2500 m and 5000 m) using R programming language (library argosfilter, function sdafilter) [25]. Although state-space based methods of filtering were attempted, due to the nature of sampling (the duty cycles employed resulted in large gaps in the data and a lack of frequent, regular locations), these methods could not successfully be applied to this data set. For further details on the impact of tag programming to the application of state-space models see [26].

### Time Spent Grids

A 100 km^2^ grid was applied to the tracking area. We calculated the total time spent by all individuals, and the total number of individuals present within each grid square. We generated two measures of occupancy. The first measure (“measure of occupancy 1”) allocated to each individual the time spent in each grid square as a proportion of the total time spent by that individual in all grid squares. These individual values were then summed to give a sum of the proportion of time spent by all individuals in all grid squares. This first measure of occupancy was highly influenced by short tracks that occur across few grid squares therefore a second measure of occupancy was also generated. The second measure (“measure of occupancy 2”) used the time spent per grid square per individual multiplied by the total time spent by that individual as a fraction of the total time spent by all individuals. This measure was then summed for all individuals across all grid squares. This second measure was influenced by long tracks placing a higher weighting on their contribution to the time spent per grid square than shorter tracks.

### Environmental and Geographic Datasets

Environmental covariates were extracted for each location across the tracking period: bathymetric data (GEBCO_08 Grid, version 20100927: http://www.gebco.net; near surface chlorophyll *a* concentration (MODIS aqua satellite, 9 km and 8 day resolution): http://oceancolor.gsfc.nasa.gov/; and sea surface temperature (0.25 degree and daily resolution): http://www.ncdc.noaa.gov/sst/.

## Results

Satellite tags were deployed on three blue whales in 2009 and 12 blue whales in 2011 within the Perth Canyon, approximately 32.0°S, 115.0°E, off western Australia. All tags deployed in 2009 transmitted successfully. However, of the 12 tags deployed in 2011, four performed very poorly providing either no transmissions at all (n = 2), no location data (tag was transmitting but did not generate locations; n = 1), or failed within a day (n = 1). Poor tag performance may possibly result from electronic failure or structural failure resulting in rapid migration of the tag out of the whale.

The remaining 11 whales were tracked from between 8 and 308 days (mean = 66.3 days, standard error = 26.1, Table 1). A total of 2793 locations (including LC Z) were received of which 1378 locations were retained post filtering. Throughout the tracking period, each satellite tagged whale covered an average distance of 3,009±892 km (mean ± se; range: 382–10,467 km) at a rate of 21.9±0.7 km per day (0.09–455.8 km).

**Table 1:** Satellite tag deployment details and movement descriptors from the filtered Argos location data.

Ptt	Sex	Deploymentdate	Firsttransmission	Lasttransmission	Trackingduration (days)	Transmittingdays	Locations/day(mean ± se; range)	Trackdistance (km)	Distance/day(mean ± se; range)
53734	Unknown	18/03/2011	18/03/2011	19/04/2011	32	29	4.06±0.56; 0–10	1649	49.52±4.91;8.54–125.49
53791	Female	30/03/2011	30/03/2011	28/04/2011	29	25	2.62±0.42; 0–8	2695	89.66±8.16;10.71–250.12
98106	Unknown	26/03/2011	28/03/2011	03/05/2011	36	13	0.42±0.11; 0–2	1826	51.98±6.44;10.26–122.78
98108	Male	30/03/2011	30/03/2011	17/05/2011	48	33	1.63±0.28; 0–8	3751	77.95±5.86;5.00–235.96
98115	Unknown	14/03/2011	14/03/2011	11/05/2011	58	32	1.08±0.17; 0–4	1631	28.93±3.05;3.47–107.28
98134	Unknown	17/03/2011	17/03/2011	06/04/2011	20	20	3.35±0.70; 0–10	1690	78.24±10.10;31.08–195.15
98135	Unknown	06/04/2011	06/04/2011	08/02/2012	308	145	1.60±0.12; 0–11	10,468	57.52±3.83;1.14–462.03
98141	Female	30/03/2011	11/06/2011	09/07/2011	28	13	0.50±0.12; 0–2	832	29.72±4.08;3.07–93.72
88731	Male	08/04/2009	08/04/2009	15/04/2009	8	7	3.75±1.32; 0–10	382	43.46±10.56;13.86–101.57
88740	Female	06/04/2009	06/04/2009	04/05/2009	29	29	4.58±0.33; 1–10	1,807	61.00±6.35;21.03–161.06
88739	Male	08/04/2009	08/04/2009	18/08/2009	133	122	2.02±0.11; 0–5	6,378	47.08±2.77;3.08–205.76

**Figure 1:**
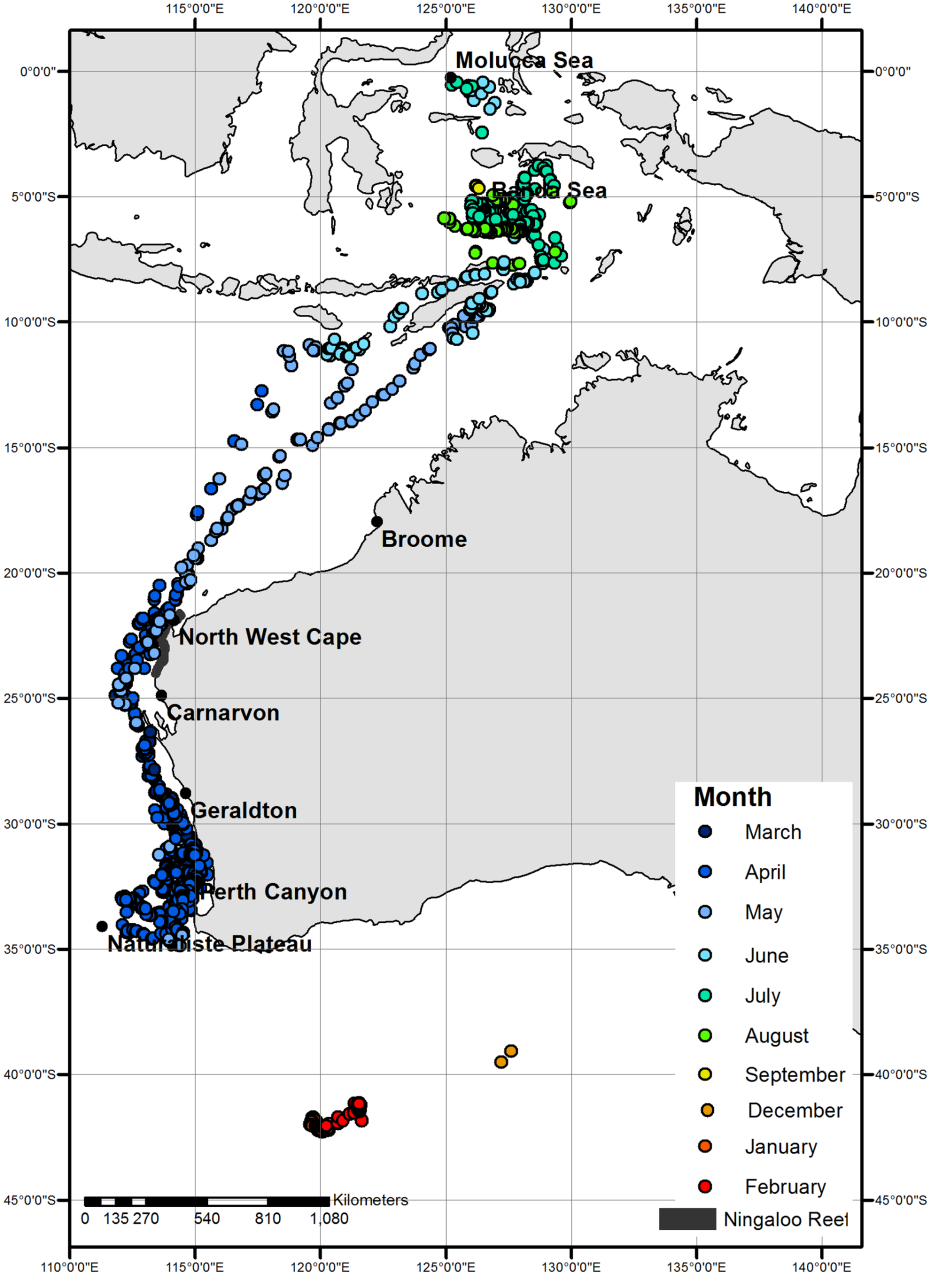
Filtered satellite tag derived locations of pygmy blue whales (n = 11) by month. Individuals were tagged in March (2011: n = 7) and April (2009: n = 3; 2011: n = 1) in the Perth Canyon. The northern terminus of migration occurred in Indonesia. A single whale was tracked intermittently until February 2012 at which time it was located in the subtropical frontal zone.

All whales were tagged during March (2011: n = 7) and April (2009: n = 3; 2011: n = 1) and ultimately migrated northwards post tag deployment (Fig. 1). Five whales (2009: n = 2; 2011: n = 3) initially travelled in a loop southwards towards the Naturaliste Plateau (34.1°S, 111.3°E) prior to a direction change north (2009: n = 3; tag ceased transmitting: n = 2) whilst another five whales (2009: n = 1; 2011: n = 4) travelled northwards immediately post tagging. One satellite tag deployed in 2011 did not initiate transmission of locations until June at which time the whale had reached the northern terminus of its migration in the Banda Sea. All 11 tracks are detailed individually within the supplementary material (Fig. S1).

**Figure 2:**
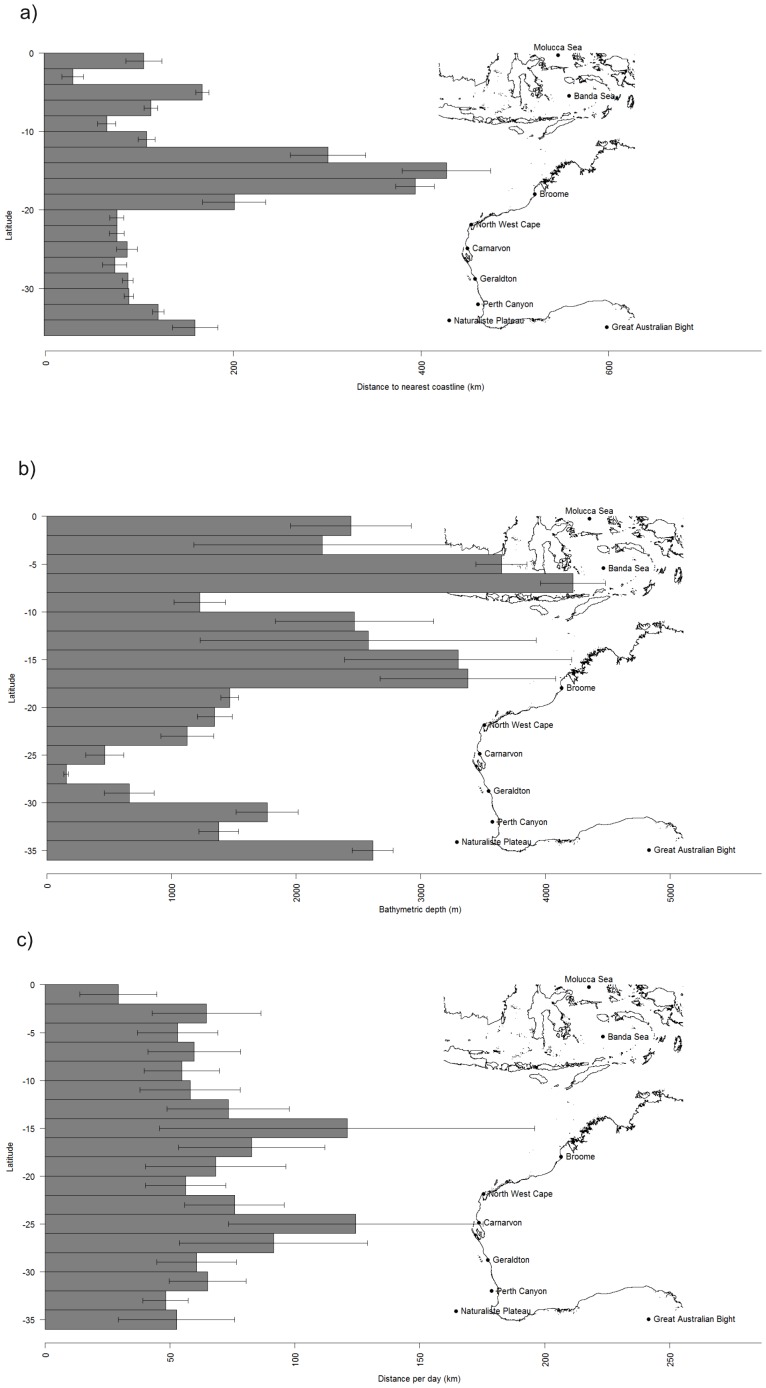
Pygmy blue whale migration (n = 11) towards Indonesia in relation to 2° bins of latitude. a) Distance to coastline (km), b) bathymetric depth (m) and c) distance travelled per day (km). All bars represent mean values with 95% confidence intervals. Map of Australia is included to indicate the position of each longitudinal bin in relation to the Australian and Indonesian coastline.

**Figure 3:**
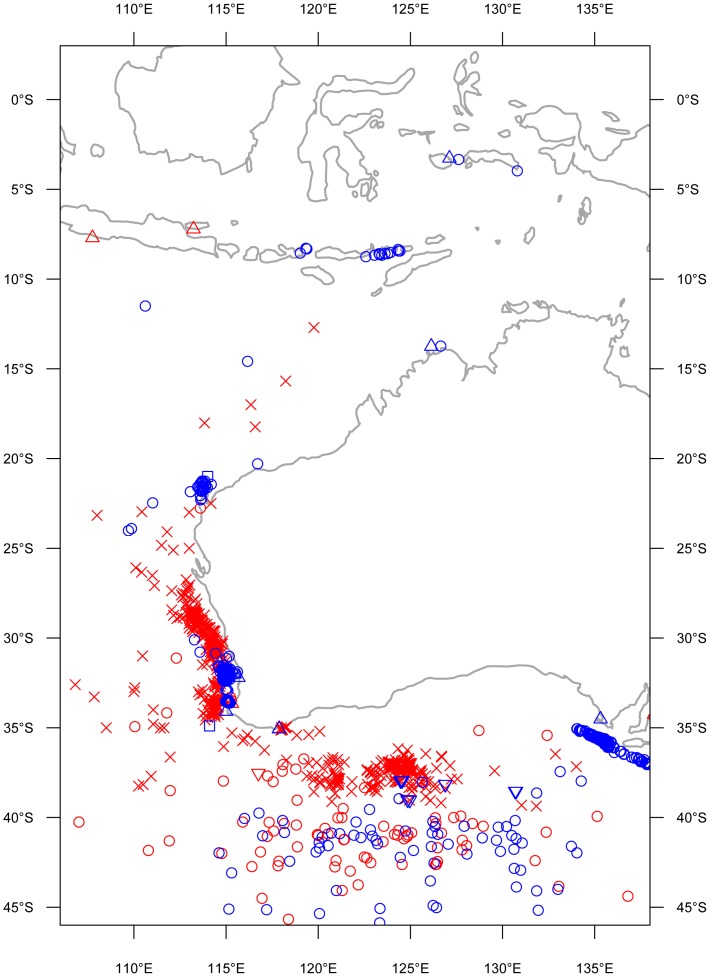
Positional information for pygmy blue whales. Catches (x), sightings (○), strandings (Δ), acoustic recordings (□) and discovery marks (∇) collected up to and including 1973 when whaling stopped (red) and after 1973 (blue). Modified from [4].

The tagged whales travelled relatively near to the western Australian coastline (100±1.7 km; Fig. 2a) throughout March (Fig. 1; 2011: n = 6) and April (Fig. 1; 2009: n = 3; 2011: n = 7) until reaching North West Cape (22.23°S, 113.96°E)–a prominent peninsula in the northwest of the Australian state of Western Australia. Animals continued to travel northwards and offshore (238.0±13.9 km; Fig. 2a) during May (Fig. 1; 2009: n = 2; 2011: n = 4) towards Indonesia. By June, whales were travelling through the Savu and Timor Seas (Fig. 1; 2009: n = 1; 2011: n = 2) and were located within 137.3±3.1 km of the nearest Indonesian coastline. The northern terminus of migration for the remaining whales was within the Banda (2009: n = 1; 2011: n = 1; 6°S, 127°E) and Molucca Seas (2011: n = 1; 0.42°S, 125.42°E), just south of the equator. Only one satellite tag (deployed in 2011) continued to relay location information after August 1^st^, locating the whale within the Banda Sea until September after which a temporary pause in location transmission occurred until December. This whale migrated southwards throughout the three months in which location information was not transmitted. Once the tag resumed transmitting, the whale was located at the approximate position of the subtropical front [27], south of western Australia for at least three months until tag failure in February (Fig. 1). These 11 satellite tracks line up closely with blue whale positional information collected both pre and post whaling including sightings, strandings, acoustic recordings and Discovery mark retrievals (Fig. 3; modified from [4]).

Pygmy blue whales occupied shallowest (1369.5±47.4 m) waters when closest to the coast throughout March and April (Fig. 2a and 2b), migrating into progressively deeper waters offshore (2617.0±143.5 m) in May and then often deep coastal Indonesian waters (3788.5±66.4 m) from June to September. Rate of travel increased slightly from 66.7±3.6 km/day in March and April to 77.0±7.4 km/day in May. Once the animals reached the northern end of the migration, rate of travel slowed to 58.3±7.4 km/day (June–September).

**Figure 4:**
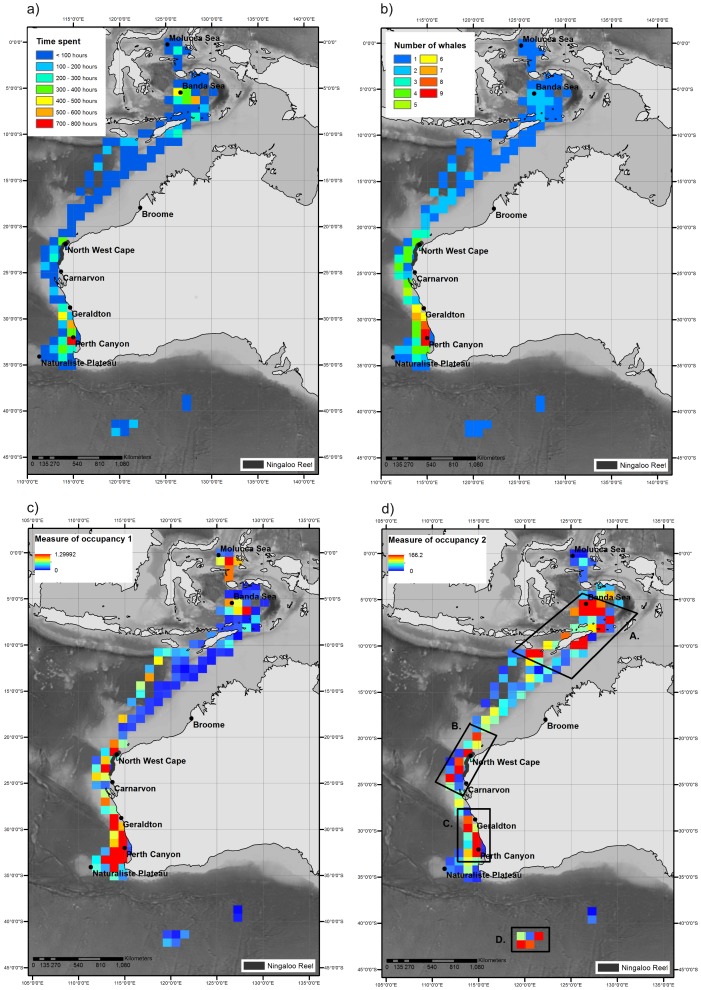
Gridded measures of time spent and occupancy for satellite tagged pygmy blue whales (n = 11). a) Total time spent, b) number of whales, c) measure of occupancy 1: proportion of time spent per grid square per individual summed across all individuals and d) measure of occupancy 2: sum of individual time spent per grid square adjusted by the contribution to total time spent by all individuals for all pygmy blue whales throughout the tracking period. Four regions of potentially higher occupancy are indentified in Fig. 4d): A. Indonesia, B. Ningaloo Reef, C. Perth Canyon/Naturaliste Plateau and D. Subtropical frontal zone. The grid presented is 100 km×100 km. GEBCO bathymetry is also shown.

When examining total time spent by all whales across the duration of the tracking period, there were areas with distinctly higher occupancy times (Fig. 4a). Even accounting for the larger number of active tags earlier in the tracking period, areas such as the Perth Canyon (single grid cell time spent: 773.8 hours – Fig. 4a; number of whales: 9– Fig. 4b), the Naturaliste Plateau region (time spent across grid cells occurring in the Naturaliste Plateau area: at least 1100 hours – Fig. 4a; number of whales: at least 4– Fig. 4b) and North West Cape (single grid cell time spent: 330.3 hours – Fig. 4a; number of whales: 4– Fig. 4b) appeared to be areas in which occupancy was relatively prolonged. Areas of high occupancy time were also found within the Banda (single grid cell time spent: 599.8 hours – Fig. 4a; number of whales: 3– Fig. 4b) and Molucca Seas (single grid cell time spent: 232.4 hours – Fig. 4a; number of whales: 1– Fig. 4b), Indonesia. Upon examination of the number of whales that occurred within each grid square (Fig. 4b), it was apparent that tracks converged around the North West Cape region whilst milling was evident in the Perth Canyon/Naturaliste Plateau region.

The first measure of occupancy highlights the importance of the tagging location - the Perth Canyon/Naturaliste Plateau region (Fig. 4c). Even though this measure was highly influenced by short tracks, the Perth Canyon/Naturaliste Plateau region was an area in which at least five of the tagged individuals milled (Fig. 1; Fig. 4b) before migrating north. By accounting for the contribution of each individual to the entire dataset, the second measure of occupancy (Fig. 4d) highlights the importance of areas through which the longer tracks traversed such as Indonesian waters and the subtropical frontal zone. To represent potential areas of higher occupancy throughout the entire tracking range therefore, we examined all areas in which the grid square value for the second measure of occupancy was greater than the mean value (mean = 48.2, equating to all dark yellow, orange and red grid squares in Fig. 4d). This resulted in the demarcation of four regions: A. Indonesia, B. Ningaloo Reef, C. Perth Canyon/Naturalist Plateau and D. Subtropical frontal zone.

**Figure 5:**
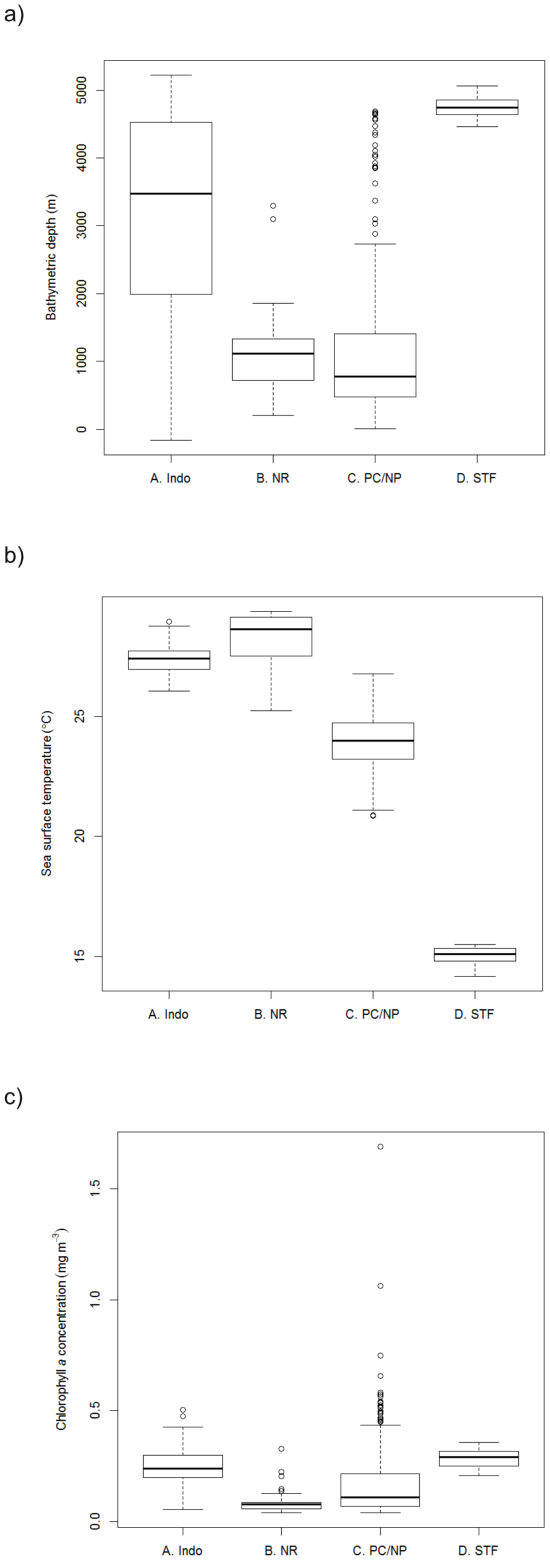
Boxplots describing the oceanographic characteristics of the four regions of potentially higher occupancy as identified in Fig. 4d. A. Indonesia (n = 324), B. Ningaloo Reef (n = 106), C. Perth Canyon/Naturaliste Plateau (n = 396) and D. Subtropical frontal zone (n = 78): a) bathymetric depth (m), b) sea surface temperature (°C) and c) chlorophyll *a* concentration (mg m^−3^).

The environmental and oceanographic properties of these four regions of potentially higher occupancy differed (Fig. 5). Individuals occupied shallow water in Ningaloo Reef (1088±47 m) and Perth Canyon/Naturaliste Plateau (1176±58 m) as compared to Indonesia (3192±80 m) and the subtropical frontal zone (4749±16 m; Fig. 5a) and occupied warmer waters off Ningaloo Reef (28.2±0.1°C) than when in Indonesia (27.3±0.03°C; Fig. 5b). Pygmy blue whales in the Indonesia region occupied waters with comparable productivity to the subtropical frontal zone (Indonesia: 0.24±0.005 mg m^−3^, subtropical frontal zone: 0.28±0.005 mg m^−3^; Fig. 5d). However, the highest values of productivity occurred in the Perth Canyon/Naturaliste Plateau region (max = 1.69 mg m^3^; Fig. 5d) with Ningaloo Reef producing the lowest productivity values (min: 0.04 mg m^−3^; Fig. 5d).

## Discussion

These satellite tag derived movements of pygmy blue whales provide important new information potentially indicating the general migration pattern and breeding grounds of pygmy blue whales that feed off the western coast of Australia. Assuming these movements are representative of the animals that feed off the western Australian area as a whole, pygmy blue whales migrate north from the Perth Canyon/Naturaliste Plateau region in March/April reaching Indonesia by June where they remain until at least September. Southern migration from Indonesia may occur from September and finish by December in the subtropical frontal zone after which the animals may make their way slowly northwards towards the Perth Canyon by March/April. This temporal pattern of migration as revealed by satellite telemetry is supported by acoustic recordings of Australian type pygmy blue whale calls off south western Australia [7]–[9], [28], [29]. However these calls have also been recorded well to the west of this proposed migratory path within Subantarctic waters [10], [11] and potentially Antarctic waters [11], [12] indicating multiple migration routes, elasticity in migratory behaviour or the potential for calls to be detected hundreds of kilometres from the source. Elasticity in migratory behaviour has been demonstrated by eastern North Pacific blue whales and is hypothesised to be related to changes in prey availability mediated by oceanographic processes [30].

The single satellite tagged whale that was tracked to the subtropical frontal zone south of Australia was about 900 km west of the other well described pygmy blue whale feeding area, the Bonney Upwelling (between 139.75°E and 143°E) [14]. This subtropical frontal zone region is also an area in which blue whales were caught and sightings recorded historically. Genetic evidence indicates mixing between the animals in the feeding areas of the Perth Canyon (off western Australia) and Bonney Upwelling (off southern Australia) [18], and animals photographed in the Bonney Upwelling have also been resighted in the Perth Canyon [15]. This indicates the potential for individuals from the Bonney Upwelling to follow a similar migration route to those animals feeding in the Perth Canyon.

When whales were migrating north, lower rates of travel and relatively higher occupancy were recorded in the Perth Canyon/Naturaliste Plateau region, North West Cape/Ningaloo Reef region, Indonesian waters and the subtropical frontal zone. It is known that feeding occurs off the Perth Canyon [13] and that the Indian Ocean sector of the subtropical frontal zone is a productive region in which phytoplankton blooms occur [31]. The North West Cape/Ningaloo Reef region has the capacity to offer feeding opportunities as primary production rates are equal to those recorded in current upwelling systems [32] and the reef possesses unique biophysical conditions able to support large biomasses of marine species [33] including other migratory krill feeding specialists [34], [35]. Indeed, surface lunge feeding of pygmy blue whales has been observed at North West Cape and Ningaloo Reef in June (C. Jenner and M-N Jenner, unpublished data, 2001). However, the low values of productivity experienced by the whales in this study indicate that the higher measure of occupancy attributed to the North West Cape/Ningaloo Reef region may be a result of tracks narrowly converging as individuals round the peninsula on their way north.

As with most baleen whales species, blue whales are thought to migrate between high and low latitudes: at high latitudes they feed and at low latitudes they calve [36], [37]. The breeding regime of pygmy blue whales caught in the waters around Kerguelen Island may offer insight into the breeding regime of pygmy blue whales occupying Australian waters considering Australian pygmy blue whale type calls have been detected as far west as the Crozet Islands [10] and around Kerguelen Island [11]. For these animals, foetal lengths and pregnancy ratios indicate that mating occurs between November and January whilst calving occurs in the austral winter with another minor calving event in the austral summer [2]. The timing of movement as suggested by the satellite tagging data presented here indicates that pygmy blue whales may calve in the Banda and Molucca Seas, highlighting the need for ongoing conservation and management efforts for blue whales in Indonesian waters.

Behavioural plasticity as a reflection of changing prey fields is common to a wide variety of marine vertebrates [38] therefore presumably pygmy blue whales are adapted to exploit widely dispersed and ephemeral temperate and tropical food sources. Archival dive data from a tag deployed on a blue whale in Indonesia between May and July provided evidence of feeding within Indonesian waters (B. Kahn, unpublished data). Our results also indicate that pygmy blue whales occupy relatively productive waters when in the Indonesia region. Frontal formation occurs within the Banda and Molucca Seas [39] and upwelling is enhanced during the south-east monsoon that occurs between May and August in the eastern Banda Sea [40] leading to high productivity throughout the time in which pygmy blue whales are located in the area. The Banda and Molucca Seas therefore may also act as a feeding ground for pygmy blue whales and have certainly been indicated as important foraging locations for another migrating marine vertebrate [41].

Throughout their migratory range, satellite telemetry demonstrates that pygmy blue whales move through areas posing multiple risks. Along the western Australian coastline, whales migrate through an area that contains the majority of Australia’s gas resources and in which production and development is ongoing [42]. As such, pygmy blue whales occupying or travelling through these areas are exposed to anthropogenic noise associated with industry, shipping and seismic exploration. In Indonesian waters, pygmy blue whales encounter fisheries related risks such as entanglement from offshore gillnets and acoustic habitat degradation from reef bombing (B. Kahn, unpublished data). Baleen whales use sound for communication and to gain information about the environment they occupy [43]. Blue whales are more likely to emit calls when in the presence of noise generated by ship traffic [44] and to increase calling rates associated with social encounters and feeding when exposed to noise generated by seismic surveys [45]. Elevated ambient noise potentially reduces an individual’s ability to detect socially relevant sounds therefore an increase in call rate is likely a compensatory behaviour to maintain communication with conspecifics in the presence of increased noise levels [44], [45].

Whilst anthropogenic noise may alter blue whale behaviour, it is unlikely to pose a conservation risk unless it causes population level consequences such as changes in growth, reproduction and survival of individuals [46]. Elevated ambient noise has been responsible for abandonment or avoidance of critical habitat by a number of cetacean species including gray whales [47], bowhead whales [48] and killer whales [49]. Critical habitat includes habitat used to meet essential life cycle requirements such as foraging and breeding (http://www.environment.gov.au/cgi-bin/sprat/public/publicregisterofcriticalhabitat.pl), both of which are activities likely to be impacted by elevated ambient noise for these pygmy blue whales. As such, displacement from critical habitat would likely impact growth, reproduction and survival. In addition, exposure to ship noise has been linked to chronic stress in right whales with physiological implications that may impact the recovery of this endangered population [50]. Whilst population level impacts such as displacement and chronic stress do pose a conservation risk, current studies of large cetaceans are unlikely to provide evidence of noise and stress directly impacting on growth, reproduction and survival due to difficulties associated with establishing long term studies of known individuals that can be predictably tracked throughout their lifetime. With additional tracking data, the potential exists to generate habitat models to describe where pygmy blue whales are found and therefore assess and mitigate anthropogenic effects. In the meantime, the description of movements as presented here can be used by managers within a precautionary principle framework to identify risks within the pygmy blue whale migratory range such as increased ambient noise from development, shipping and fishing and therefore assist in mitigating these risks. For industry, these movements are invaluable when examining potential interactions with pygmy blue whales and will enable informed decisions regarding aspects of development such as vessel traffic, seismic exploration and field locations. A combined approach by industry and managers when accounting for the movements of the pygmy blue whales utilising Australian waters will ensure the ongoing recovery of this previously exploited species.

## Supporting Information

Figure S1 **Filtered satellite tag derived locations of pygmy blue whales by month.** a) 53734, b) 53791, c) 88731, d) 88739, e) 88740, f) 98106, g) 98108, h) 98115, i) 98134, j) 98135, k) 98141, l) all tracks.
(PDF)
