# Supplementary figures and images for: Migratory Movements of Pygmy Blue Whales (Balaenoptera musculus brevicauda) between Australia and Indonesia as Revealed by Satellite Telemetry
Source: PLoS One. 2014 Apr 9;9(4):e93578. doi: 10.1371/journal.pone.0093578 (PMC3981711; doi:10.1371/journal.pone.0093578)

a)

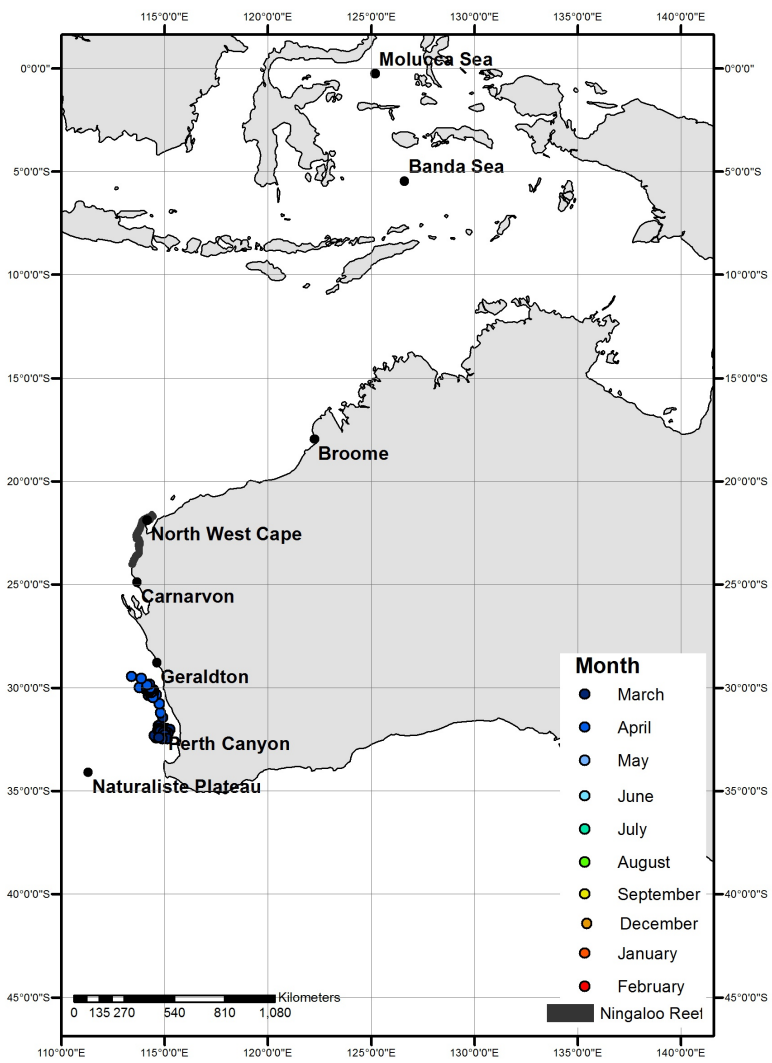

b)

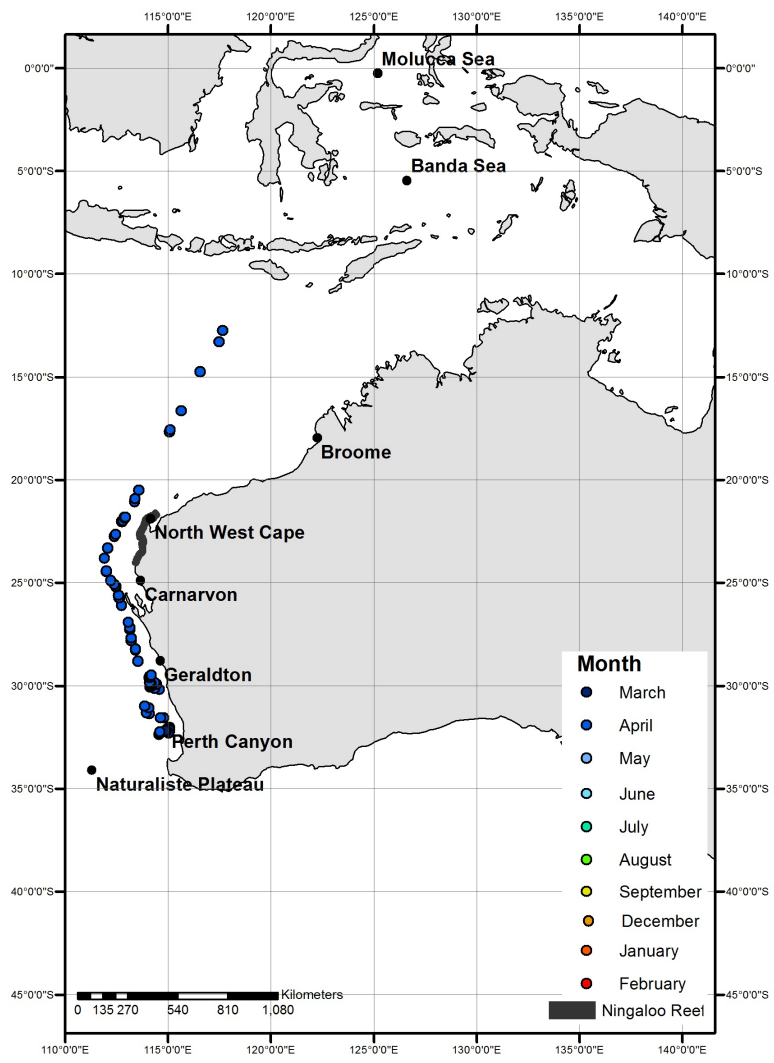

c)

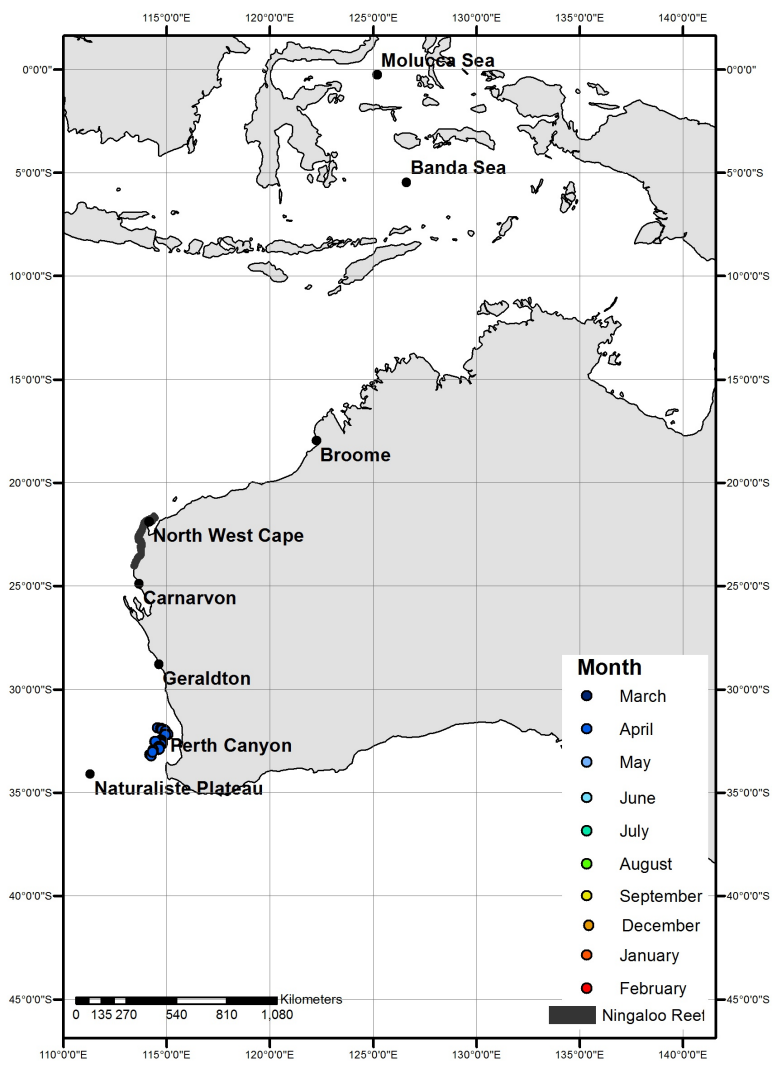

d)

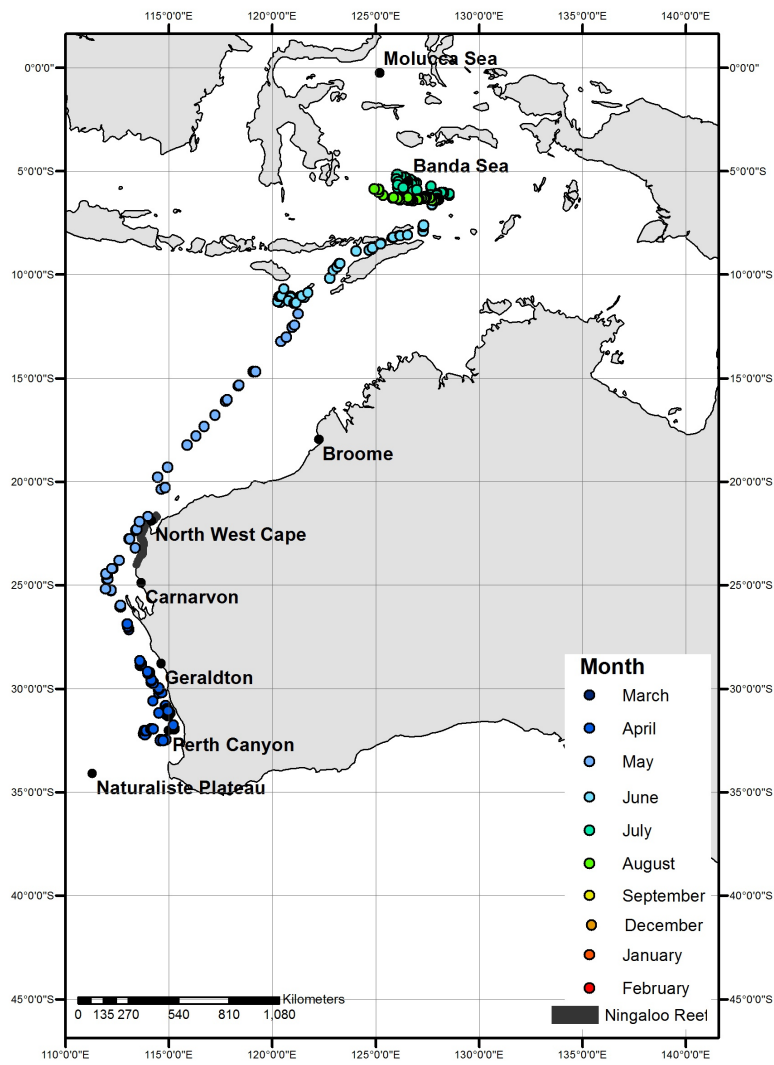

e)

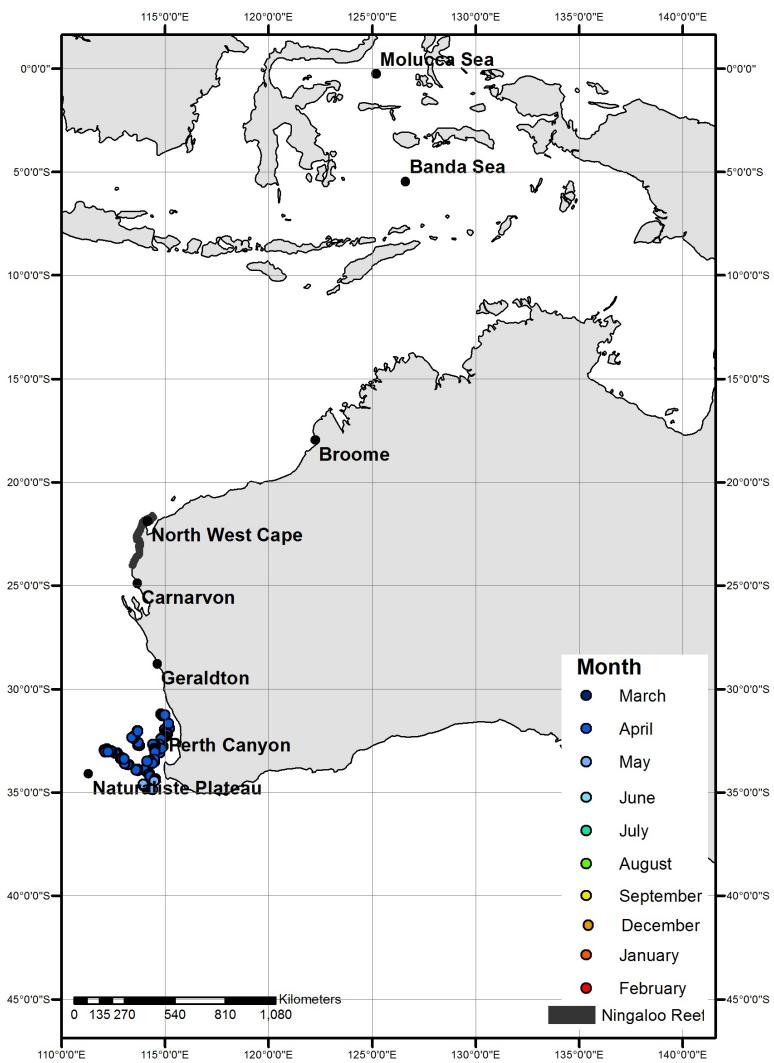

f)

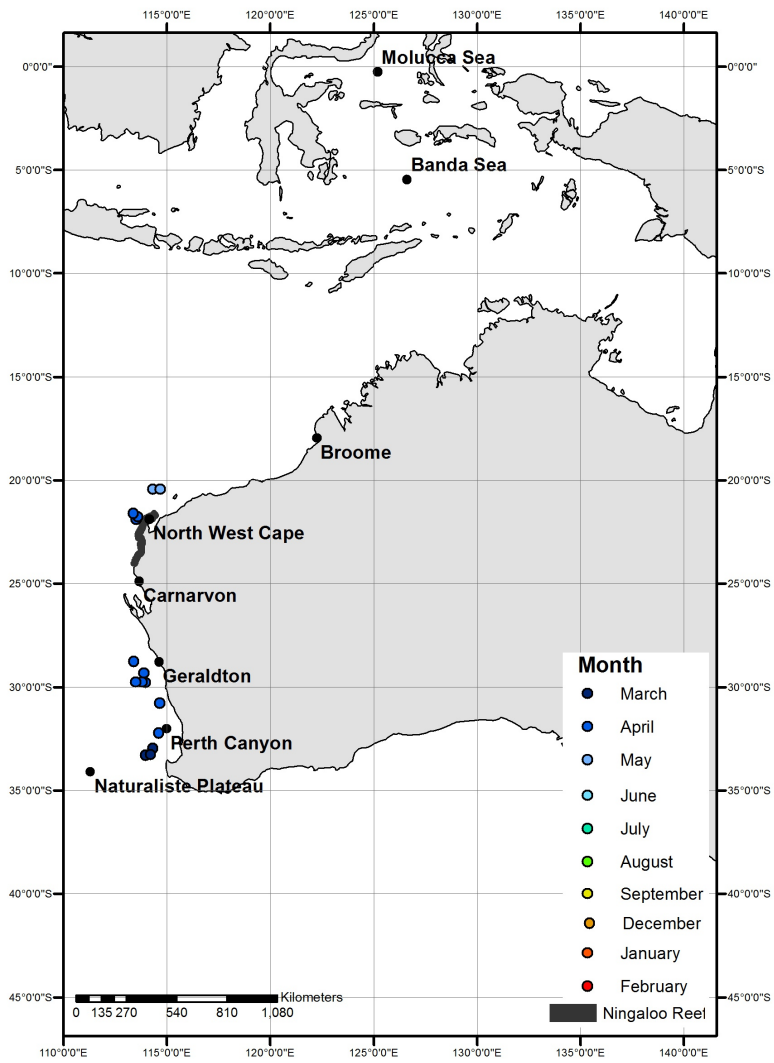

g)

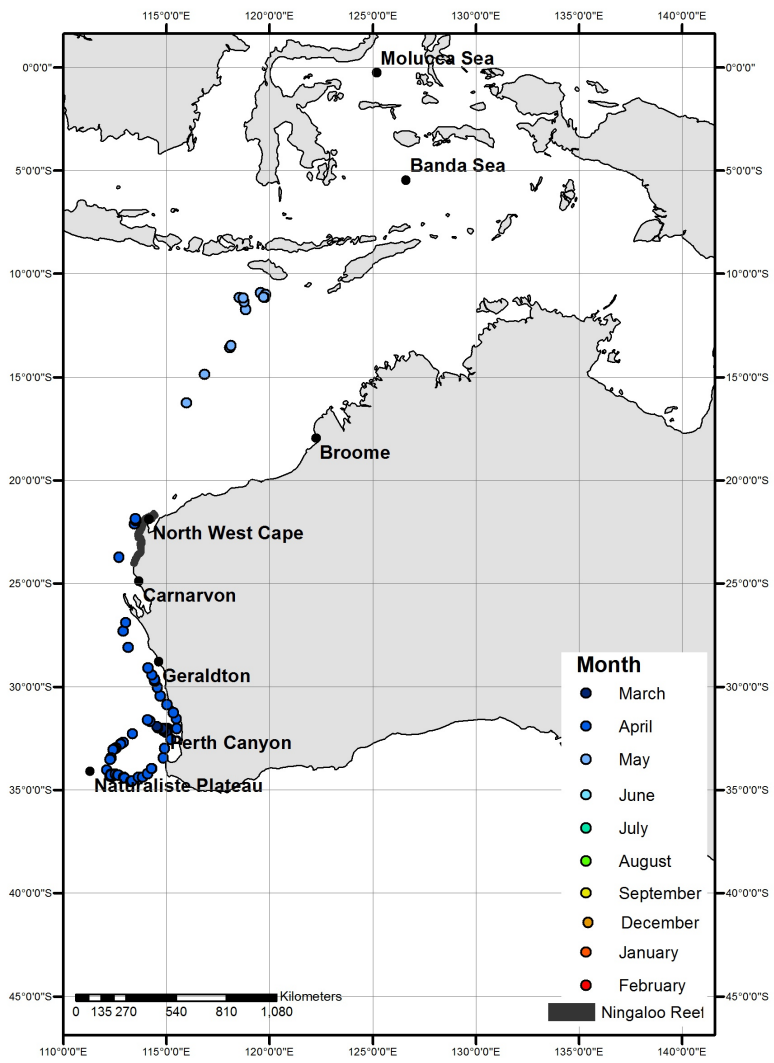

h)

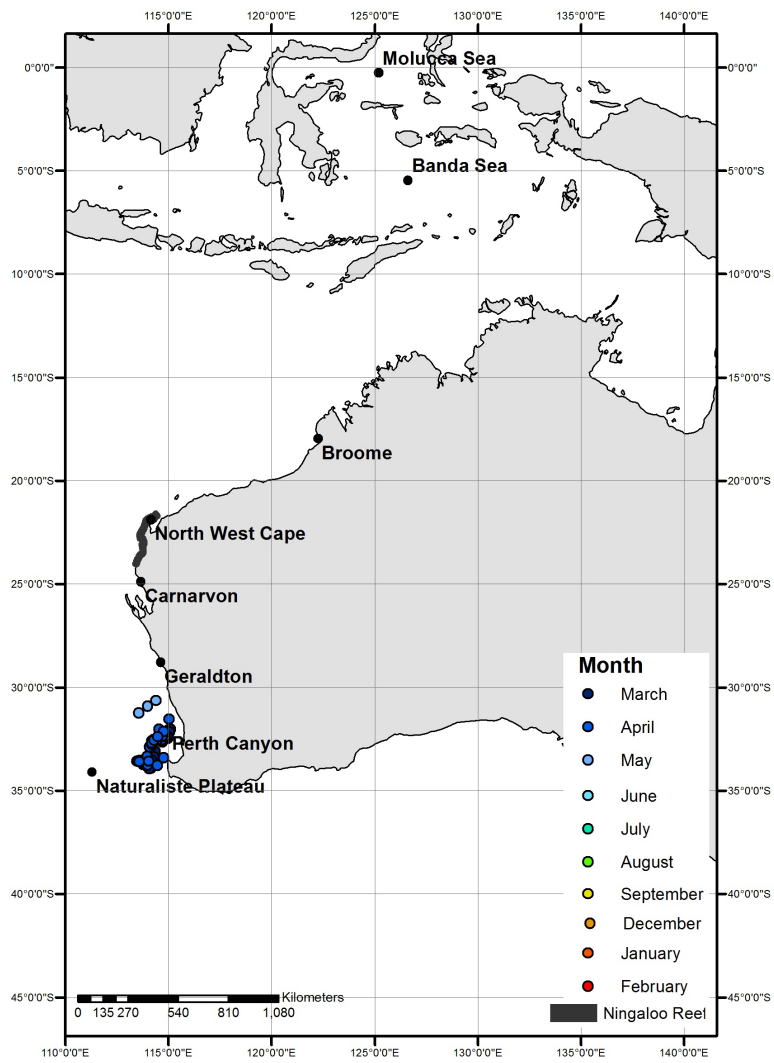

i)

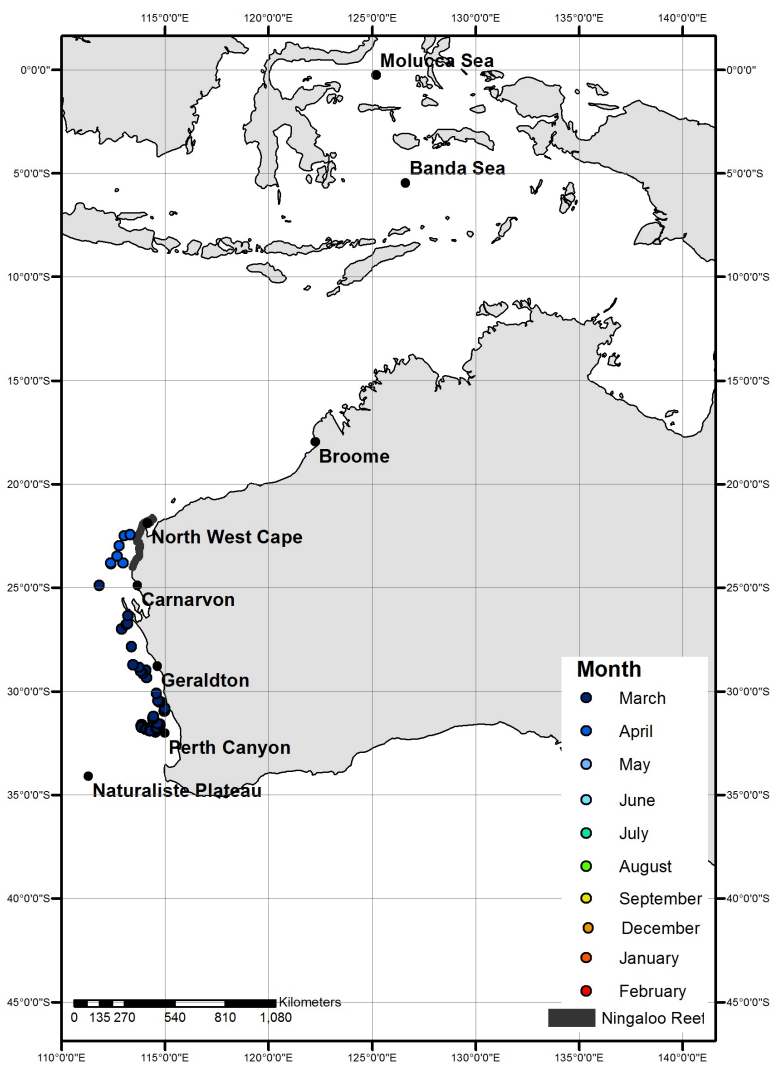

j)

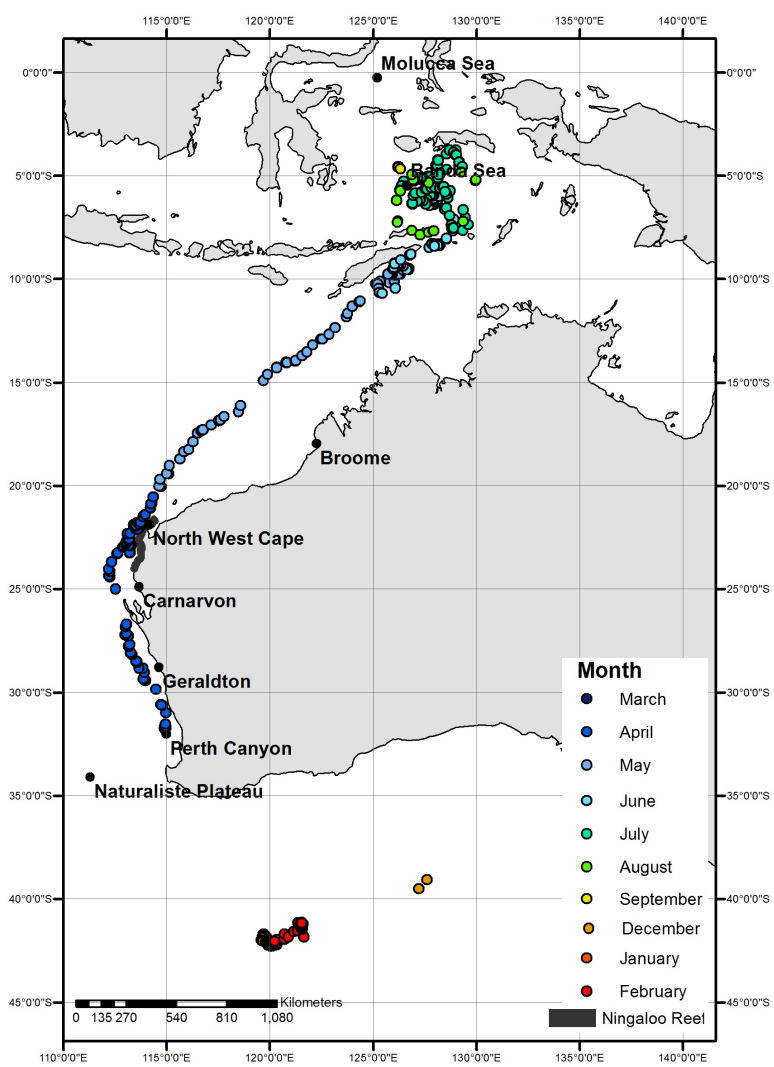

k)

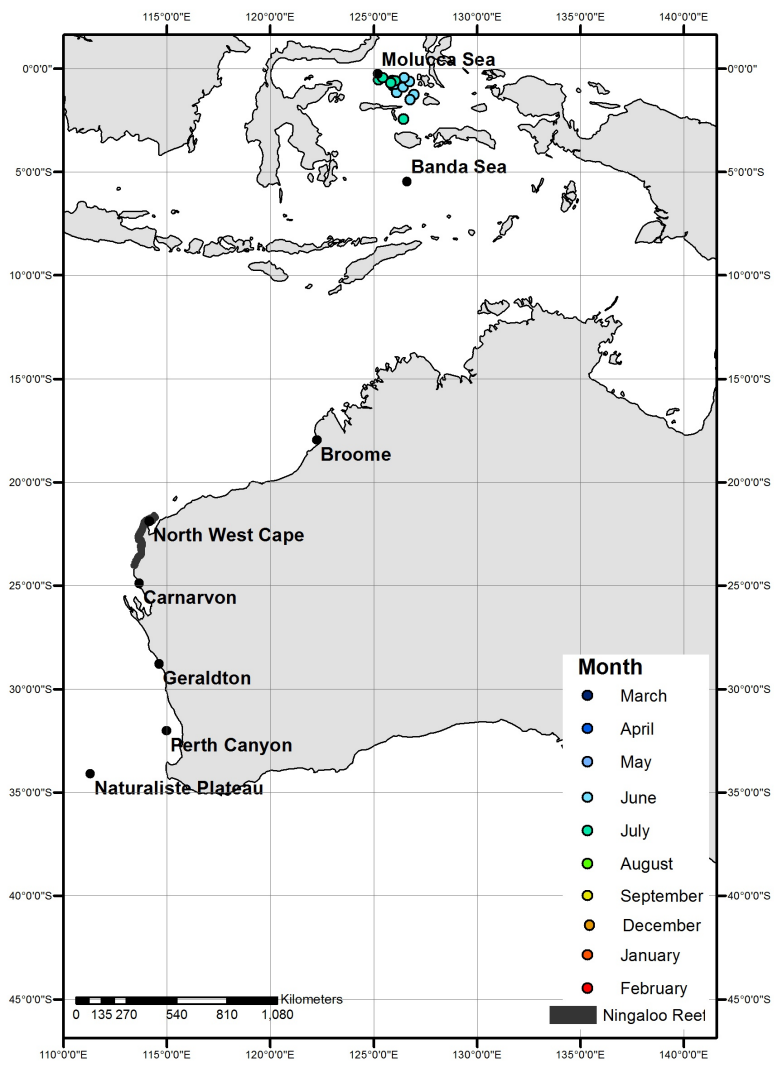

l)

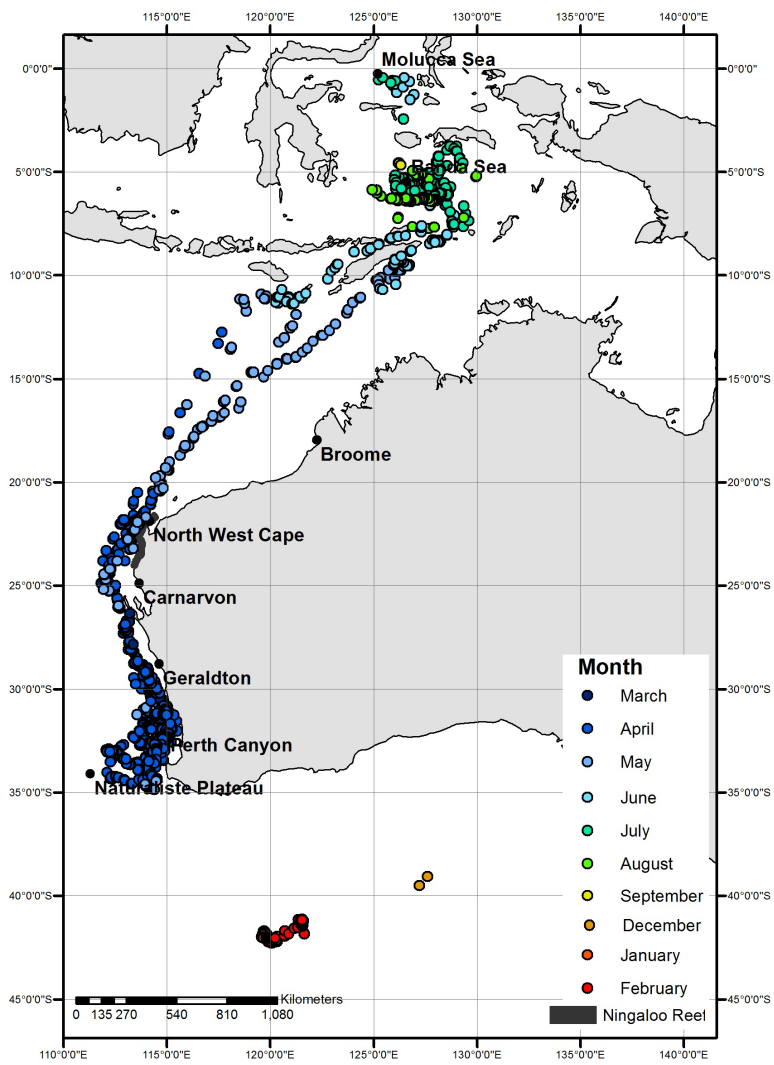

Supplement: Figure S1 — Filtered satellite tag derived locations of pygmy blue whales by month. a) 53734, b) 53791, c) 88731, d) 88739, e) 88740, f) 98106, g) 98108, h) 98115, i) 98134, j) 98135, k) 98141, l) all tracks. (PDF) [file pone.0093578.s001.pdf]
